# Effect of online education on the knowledge on, attitudes towards, and skills in patient safety for nursing students in Korea: a mixed-methods study

**DOI:** 10.3352/jeehp.2022.19.14

**Published:** 2022-06-30

**Authors:** Dan Bi Cho, Won Lee, So Yoon Kim, Sungkyoung Choi

**Affiliations:** 1 Department of Medical Law and Bioethics, Yonsei University, Seoul, Korea; 2 Department of Nursing, Chung-Ang University, Seoul, Korea; 3 Department of Medical Law and Bioethics, Yonsei University College of Medicine, Seoul, Korea; 4 Department of Nursing, Catholic Kwandong University, Gangneung, Korea; Hallym University, Korea

**Keywords:** Patient safety, Nursing education, Nursing students, Distance education, COVID-19

## Abstract

**Purpose:**

The purpose of this study was to evaluate the impact of synchronous online education on the patient safety competency (knowledge, attitudes, and skills) of nursing students in Korea and to explore what they thought about this educational method.

**Methods:**

A single-group pre-and post-test design and summative content analysis were implemented. On November 14th, 2020, 110 nursing students completed synchronous online patient safety education. Patient safety competency was measured before and after the intervention using the revised Patient Safety Competency Self-Evaluation tool. The descriptive statistics, paired t-test, and Wilcoxon signed-rank test were used to analyze the data. Students also expressed their opinions about this education based on open-ended questions.

**Results:**

All the patient safety competency scores significantly increased after intervention. A summative content analysis of the open-ended questions yielded 5 categories: educational materials, education level, education time, interaction, and educational media.

**Conclusion:**

This study found that synchronous online patient safety education improves nursing students’ knowledge on, attitudes towards, and skills in patient safety. Nursing students also expressed a variety of positive aspects of the online education method. To improve the efficacy of synchronous online patient safety education, there is a need for further empirical studies on the appropriate class duration and difficulty of the content. It is essential to find a way to combine online education with various learning activities.

## Introduction

### Background/rationale

Coronavirus disease 2019 (COVID-19) has generated awareness about patient safety, such as the need to prevent infection, and has further emphasized the importance of nurturing nurses who can professionally respond to patient safety issues [1]. To prevent infection, traditional teaching methods have quickly been replaced with the online format [2]. However, studies on the effectiveness of online patient safety education are still insufficient. Synchronous online education allows students to engage at fixed times in different physical locations, which helps students maintain tension related to learning and class participation [3]. A meta-analysis on the learning effects showed that synchronous online education was more effective for knowledge promotion than both asynchronous online and face-to-face education [4].

### Objectives

It aims to evaluate the effects of synchronous online education on nursing students’ competency, including knowledge, attitudes, and skills, and to investigate what nursing students think of this educational method. We hypothesized that synchronous online patient safety education has a positive effect on nursing students’ knowledge of, attitudes toward, and skill of patient safety.

## Methods

### Ethics statement

This study was approved by the Institutional Review Board of Severance Hospital (Y-2020-0067). Informed consent was obtained from all participants.

### Study design

Quantitative and qualitative research were used to achieve 2 separate research objectives. For quantitative research, a single-group pre- and post-test study was conducted, while for qualitative research, a summative content analysis was conducted. The manuscript was described according to the Strengthening the Reporting of Observational Studies in Epidemiology statement (https://www.strobe-statement.org) for quantitative study and Consolidated Criteria for Reporting Qualitative Research (https://www.equator-network.org/reporting-guidelines/coreq/) for qualitative study.

### Setting

Participants who agreed to participate in this study were asked to complete pre-test questionnaires. All participants who responded to the pre-test questionnaires were given a Zoom link to participate in the synchronous online patient safety education. On November 14, 2020, participants voluntarily accessed the Zoom link. We explained the research process to participants at Zoom. We also informed participants that if they had any questions during the education, they could ask questions via Zoom Chat. Two research team members (S.C. and D.B.C.), who were involved in the development of the patient safety curriculum and were well acquainted with this research, stayed in Zoom Chat during the education. Participants were asked to complete the post-test questionnaires within 12 hours after the conclusion of the education.

### Intervention

The curriculum was developed by the research team in 2019 based on the World Health Organization guidelines [5], and consisted of 7 modules, including the basic concepts of patient safety (Table 1). The instructors consisted of 5 professors majoring in medical, nursing, health science, or human engineering with expertise in patient safety; 3 board members of the Korean Society for Patient Safety (KSPS), 1 research team member and KSPS board member (W.L.), and 1 research team member (S.C.). Because unpredictable technical accidents can occur when online education is delivered in real time, the instructors were asked to record the lectures to minimize the possibility of mishaps. The lecture videos for the 7 modules on patient safety were presented live in real time for 210 minutes according to the timetable on November 14, 2020.

### Participants

**Fig. 1.**
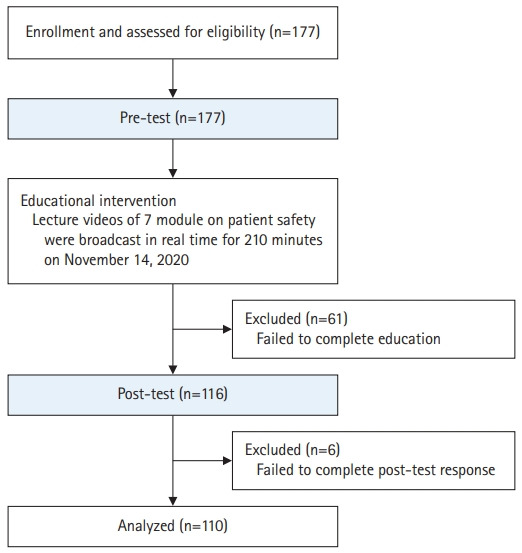
Study flow diagram

We used purposive sampling to recruit participants, who were required to meet the following eligibility criteria: frist, be a nursing student; second, understand the study aims and procedures; and third, voluntarily agree to participate. In October 2020, we distributed advertisements to 5 nursing professors among the board members of KSPS, requesting that their departments take notice of the participant recruitment. Nursing students who noticed the advertisements and agreed to participate in the study registered for the study using the online link provided in the advertisement. The recruitment period was from October 28 to November 11, 2020. A total of 177 students were initially registered, and those who failed to complete the education or who did not respond to the post-test questionnaire were excluded from the data analysis (Fig. 1).

### Quantitative study

#### Variables

We measured patient safety competency using the Patient Safety Competency Self-Evaluation (PSCSE) tool, which was developed to measure patient safety competency among nursing students in Korea [6]. We received approval from the original author to use the tool and revised some items for better applicability to the study purpose through several discussions with experts on patient safety (Supplement 1). Ultimately, the revised PSCSE tool consisted of 34 items across 3 domains, including attitudes (14 items), skills (12 items), and knowledge (8 items). All items were answered on a 5-point Likert scale, ranging from 1 (completely disagree) to 5 (totally agree). The Korean version of PSCSE had a total correlation of 0.91 [6]. In this study, the overall Cronbach’s α was 0.92, indicating the high internal consistency of the instrument. Cronbach’s α values for the sub-dimensions are as follows: attitude, 0.72; skill, 0.95; and knowledge, 0.90.

#### Data sources/measurement

Participants responded to self-administered questionnaires online, through Google Forms. Those who completed the educational intervention and pre and post-test questionnaire were given a drink coupon worth approximately US $9. They also received a completion certificate upon finishing their education. We calculated the mean values for each patient safety competency, including attitude, skill, and knowledge, and analyzed them based on grade, and learning experiences about patient safety.

#### Bias

All nursing students who met the inclusion criteria and agreed to participate in this study were included. Thus, there may have been no selection bias.

#### Study size

The sample size was calculated using G*Power ver. 3.1.9.6 (Heinrich-Heine-Universität Düsseldorf, Düsseldorf, Germany; http://www.gpower.hhu.de/) [7]. A power analysis revealed that at least 27 participants were necessary, with a medium effect size of 0.50 [8] and a power of 0.80 (P-value significant at 0.05). The actual sample size was 110.

#### Statistical methods

We summarized the general participant characteristics using descriptive statistics and used the Wilcoxon signed-rank test and paired t-test to identify differences in patient safety competency scores before and after the intervention. We analyzed all data with the SAS software ver. 9.4 (SAS Institute Inc., Cary, NC, USA). All significance testing was two-sided at the 0.05 level.

### Qualitative study

#### Personal characteristics of the research team

Research team consisted of experts on patient safety. S.Y.K. is a PhD in public health, a doctor, and a professor at the School of Medicine. She has many years of experience working on research projects in patient safety and is a principal investigator in this study. S.C. and W.L. are nurses and had PhD in public health. They have been conducting researches related to patient safety. D.B.C. is a PhD candidate. She served as a research assistant in this study.

#### Relationship with participants

Some of the researchers are nurses, so they are familiar with the nursing curriculum. Thus, they were able to provide insight into the participants’ responses to the open-ended questions. In order to minimize the impact of bias in the data analysis process, the researchers continued to discuss and exchange opinions.

#### Theoretical framework

We assessed responses qualitatively to the open-ended questions through a summative content analysis, which enriches the interpretation of the content based on the frequencies of words or manifest contents of textual data [9].

#### Data collection

The post-test questionnaire contained open-ended questions to ascertain participant perspectives on the synchronous online patient safety education intervention, as follows: first, how was the synchronous online patient safety education you received? and second, what should be supplemented to enhance the synchronous online patient safety education?

#### Data analysis

Two researchers with expertise in qualitative research and patient safety read all responses multiple times independently to identify meaningful words or contents (S.C. and D.B.C.). The codes, identifying meaningful words or content, were then grouped into one category according to similarities in the content. Frequency calculations were performed for the codes belonging to each category. All processes involved repeated discussions until consensus was gained among members of the research team.

## Results

### Participants

A total of 110 participants completed the educational intervention (Dataset 1). The mean participant age was 22.2±4.8 years (one missing data in the age variables, n=109) and 99 (90.0%) of the participants were women. For the grade, the highest proportion was 39 first-year students (35.4%), followed by 33 second-year students (30.0%). Notably, 85 participants (77.3%) had not received patient safety education prior to study engagement.

### The effects of synchronous online patient safety education

Table 2 shows changes in patient safety competency among participants both before and after the educational intervention. Before the intervention, the attitude mean of nursing students was the highest score at 4.3±0.3, followed by skill and knowledge means of 3.7±0.9 and 3.0±0.9, respectively. After the intervention, the mean scores for all the patient safety competency domains significantly increased. All the patient safety competency scores increased for year 3 and 4 student groups. Among the year 1 and 2 groups, the skill and knowledge scores were statistically increased, but there was no statistically significant difference in attitude. The scores of skills and knowledge significantly increased after the intervention, regardless of whether participants had previous experience with patient safety education. However, there were no statistically significant differences in attitude scores.

### How participants perceived synchronous online patient safety education

Sixty-five responses to open-ended questions were analyzed. We analyzed the responses, derived 115 codes, and identified five categories: educational materials; education level; education time; interaction; and educational media.

#### Educational materials

Participants said the real-world examples or videos regarding patient safety helped them understand the concept of patient safety easily (n=8). Some recommended distributing printed materials for effective synchronous online patient safety education (n=6).

#### Education level

Participants said that the instructors with expertise in the field of patient safety explained the concepts easily (n=5), and the content of the education was at a level suitable for understanding through online education as a ‘basic course on patient safety (n=6).’

#### Education time

Four people thought that 3 hours was suitable for learning the basic concept of patient safety, while 6 people felt it was too long for online education.

#### Interaction

Five participants regretted that the education was conducted only in the lecture-style method, which is mainly a one-way method of communication. Participants said that linking experience-based training, such as clinical practice, role-playing, and/or team projects, with online education would help them better understand patient safety (n=6).

#### Educational media

Ten participants positively evaluated ‘the real-time broadcast after pre-recording method’ in terms of not worrying about the scheduled time being exceeded. On the other hand, technical issues (inconsistent audio volume, system freezing, and video choppiness; n=11) were pointed out as one of the factors that disturbed concentration.

## Discussion

### Key results

This study produced empirical evidence on the effectiveness of online patient safety education for nursing students and nursing students’ perceptions towards online patient safety education. This study shows that online patient safety education improves the patient safety competency of nursing students. In addition, nursing students generally positively accepted the online patient safety education method. In other words, our analysis suggests that online education is helpful for nursing students to familiarize themselves with the concepts of patient safety.

### Interpretation

Using the revised PSCSE tool, we evaluated nursing students’ patient safety competency. Before educational intervention, knowledge competency was lower than attitudes and skills, and it can be inferred that this was due to the opportunity to learn about patient safety in the existing nursing curriculum is not enough. After educational intervention, nursing students’ relatively lower competency in skills and knowledge improved. This shows that the online patient safety education in this study can improve balanced students’ patient safety competency. The reason that there was no significant difference in attitude can be interpreted as the fact that the attitude score was already quite high before the educational intervention. According to the curriculum of nursing colleges in Korea, clinical practice begins in the third year. The results of this study showed that all 3 competencies of patient safety improved in year 3 and 4 student groups. This suggests that patient safety education is more effective when lecture-oriented conceptual learning and field-oriented learning are combined. Seventy-two (65.5%) of participants were students in lower grades who had entered the university after the COVID-19 outbreak. They were already familiar with such online education because they have only received online education since they entered the university. Therefore, the students were able to participate in the online patient safety education without hesitation, and it seems that they generally evaluated this education method positively. Participants in this study expressed both positive and negative opinions about the 3-hour class duration. The class duration should be set in consideration of the target of education and the difficulty of the content. In this study, basic concepts of patient safety were taught to undergraduate nursing students. As there are relatively few empirical studies on the optimum length of time for synchronous online patient safety education, continued research is needed.

### Comparison with previous studies

Gleason et al. [10] found that the patient safety competencies of health professionals who completed the patient safety course in massive open online courses (MOOCs) were improved. However, some experts question the effectiveness of such asynchronous online education. For example, the completion rate of MOOCs is extremely low. High self-regulatory abilities of students are essential for asynchronous online education to be implemented effectively [11]. This study shows that online patient safety education has positive effects in both synchronous and asynchronous learning. Synchronous learning means that students are connected using the same platform in real time, and focuses on ‘Time’ [12]. There are some studies on the positive aspects of nursing education using synchronous online education. Chipps et al. [13] found no differences in learning satisfaction between a face-to-face class and synchronous videoconferencing class provided to nurses in rural hospitals. Claman [14] found that family nurse practitioner students were more actively engaged in synchronous learning platforms when compared to their asynchronous counterparts.

### Limitations

There was no control group to compare the responses. Further, it is difficult to deeply interpret the meanings of qualitative responses unless participants describe their thoughts in detail.

### Generalizability

We believe it was meaningful to implement mixed synchronous and pre-recorded forms of online education as 1 method of delivering patient safety education and assisting in the development of patient safety competency for nursing students in Korea.

### Suggestions

Synchronous online patient safety education linked to role-playing, clinical practice, and team projects can help develop the competency of nursing students. Nevertheless, further investigations are needed into the relationship between proficiency in technology use and educational effectiveness in online education.

### Conclusion

The above results show that synchronous online patient safety education has a positive effect on nursing students’ knowledge of, attitudes toward, and skills in patient safety. It means that our hypothesis is accepted. The nursing students who participated in the study also evaluated the synchronous online patient safety education positively. However, studies on the effects of synchronous online patient safety education are lacking. To increase the efficacy of online patient safety education, researchers should continue to gather empirical evidence on the appropriate class duration and difficulty of the content. It is also necessary to find a teaching method that connects online education with various learning activities.

## Figures and Tables

**Table 1.** Timetable of online patient safety education

Time	Contents	Association with WHO curriculum guide (WHO, 2011)
12:30-14:15 (105’)	Module 1. Introduction to patient safety (25’)	Topics 1, 2, 5
	Module 2. Human factor engineering and systematic approach (45’)	Topics 2, 3
	Module 3. Patient safety law and policy (35’)	None
14:15-14:25 (10’)	Break time	-
14:25-16:10 (105’)	Module 4. Managing patient safety (25’)	Topics 5, 6, 7
	Module 5. Patient safety communication (15’)	Topics 4, 6
	Module 6. Engaging with patients (30’)	Topics 6, 8
	Module 7. Coping with patient safety incidents (35’)	Topics 6, 7, 8

WHO, World Health Organization.

**Table 2.** Effects of synchronous online patient safety education

Group	Domain	Pre-test	Post-test	P-value
Total (n=110)	Attitude^a)^	4.3±0.3	4.3±0.2	0.025
	Skill^b)^	3.7±0.9	4.4±0.6	<0.001
	Knowledge^b)^	3.0±0.9	4.5±0.6	<0.001
Grade				
First (n=39)	Attitude^b)^	4.3±0.2	4.3±0.2	0.764
	Skill^b)^	3.4±1.0	4.2±0.7	<0.001
	Knowledge^b)^	2.7±0.8	4.4±0.6	<0.001
Second (n=33)	Attitude^b)^	4.3±0.4	4.3±0.2	0.764
	Skill^b)^	3.7±0.7	4.5±0.4	<0.001
	Knowledge^b)^	3.2±0.9	4.6±0.4	<0.001
Third (n=29)	Attitude^a)^	4.3±0.2	4.4±0.1	0.018
	Skill^b)^	3.9±0.9	4.4±0.6	0.001
	Knowledge^b)^	2.9±1.0	4.5±0.6	<0.001
Fourth (n=9)	Attitude^b)^	4.2±0.2	4.4±0.3	0.044
	Skill^b)^	3.8±0.4	4.4±0.3	<0.001
	Knowledge^b)^	3.6±0.7	4.6±0.4	0.001
Learning experience about patient safety				
Yes (n=25)	Attitude^b)^	4.3±0.2	4.4±0.2	0.210
	Skill^a)^	4.0±0.7	4.5±0.5	<0.001
	Knowledge^b)^	3.2±1.1	4.6±0.4	<0.001
No (n=85)	Attitude^a)^	4.3±0.3	4.3±0.2	0.080
	Skill^b)^	3.5±0.9	4.3±0.6	<0.001
	Knowledge^b)^	2.9±0.9	4.5±0.6	<0.001

Values are presented as mean±standard deviation.

a) By Wilcoxon signed rank test.

b) By paired t-test.

## Footnotes

**Data availability**
Data files are available from Harvard Dataverse: https://doi.org/10.7910/DVN/SSQDDL Dataset 1. Raw response data from 110 participants to survey questionnares.

## Supplementary materials

Data files are available from Harvard Dataverse: https://doi.org/10.7910/DVN/SSQDDL

Supplement 1. Revised Patient Safety Competency Self-Evaluation (PSCSE) tool for health professional students.

Supplement 2. Audio recording of the abstract.

## References

[1] Morin KH (2020). Nursing education after COVID-19: same or different?. J Clin Nurs.

[2] Singh HK, Joshi A, Malepati RN, Najeeb S, Balakrishna P, Pannerselvam NK, Singh YK, Ganne P (2021). A survey of E-learning methods in nursing and medical education during COVID-19 pandemic in India. Nurse Educ Today.

[3] Correia AP, Liu C, Xu F (2020). Evaluating videoconferencing systems for the quality of the educational experience. Distance Educ.

[4] Ebner C, Gegenfurtner A (2019). Learning and satisfaction in webinar, online, and face-to-face instruction: a meta-analysis. Front Educ.

[5] World Health Organization (2011). Patient safety curriculum guide: multi-professional edition [Internet]. https://www.who.int/publications/i/item/9789241501958.

[6] Lee NJ, An JY, Song TM, Jang H, Park SY (2014). Psychometric evaluation of a patient safety competency self-evaluation tool for nursing students. J Nurs Educ.

[7] Faul F, Erdfelder E, Lang AG, Buchner A (2007). G*Power 3: a flexible statistical power analysis program for the social, behavioral, and biomedical sciences. Behav Res Methods.

[8] Cohen J, Cohen J (1988). Statistical power analysis for the behavioral sciences.

[9] Hsieh HF, Shannon SE (2005). Three approaches to qualitative content analysis. Qual Health Res.

[10] Gleason KT, Commodore-Mensah Y, Wu AW, Kearns R, Pronovost P, Aboumatar H, Dennison Himmelfarb CR (2021). Massive open online course (MOOC) learning builds capacity and improves competence for patient safety among global learners: A prospective cohort study. Nurse Educ Today.

[11] Pursel BK, Zhang L, Jablokow KW, Choi GW, Velegol D (2016). Understanding MOOC students: motivations and behaviours indicative of MOOC completion. J Comput Assist Learn.

[12] World Bank’s EdTech Team (2020). Remote learning, distance education and online learning during the COVID19 pandemic.

[13] Chipps J, Brysiewicz P, Mars M (2012). A systematic review of the effectiveness of videoconference-based tele-education for medical and nursing education. Worldviews Evid Based Nurs.

[14] Claman FL (2015). The impact of multiuser virtual environments on student engagement. Nurse Educ Pract.

