# Supplementary material for: Effect of online education on the knowledge on, attitudes towards, and skills in patient safety for nursing students in Korea: a mixed-methods study
Source: J Educ Eval Health Prof. 2022 Jun 30;19:14. doi: 10.3352/jeehp.2022.19.14 (PMC9478472; doi:10.3352/jeehp.2022.19.14)
Supplement: Supplementary file 2 — Supplement 1. Revised Patient Safety Competency Self-Evaluation (PSCSE) tool for health professional students. [file jeehp-19-14-suppl.docx]

**Supplement 1.** Revised Patient Safety Competency Self-Evaluation (PSCSE) tool for health professional students

**I. Patient safety attitude**

| No. | Survey question | Strongly disagree | Disagree | Neutral | Agree | Strongly agree |
| --- | --- | --- | --- | --- | --- | --- |
| 1 | Making errors in health care is preventable | 1 | 2 | 3 | 4 | 5 |
| 2 | Health care professionals should make an effort to improve patient safety | 1 | 2 | 3 | 4 | 5 |
| 3 | Health care professionals should not tolerate uncertainty in patient care | 1 | 2 | 3 | 4 | 5 |
| 4 | Health professional students need to learn about patient safety in the undergraduate courses | 1 | 2 | 3 | 4 | 5 |
| 5 | Health care professionals should routinely share information about patient safety incidents and what caused them | 1 | 2 | 3 | 4 | 5 |
| 6 | Patient safety is a high priority to health care professionals | 1 | 2 | 3 | 4 | 5 |
| 7 | Health care professionals should routinely report when certain patient safety incidents occur | 1 | 2 | 3 | 4 | 5 |
| 8 | Health care professionals should disclose errors to an affected patient and his or her family | 1 | 2 | 3 | 4 | 5 |
| 9 | If there is no harm to the patient, there is no need to report a patient safety incident | 1 | 2 | 3 | 4 | 5 |
| 10 | If I saw a patient safety incident caused by myself or others, I would keep it to myself | 1 | 2 | 3 | 4 | 5 |
| 11 | Technology and information management tools (e.g., bar codes, electronic medical record, automatic alerts and alarms, Drug Utilization Review) should be used appropriately to support safe processes of care | 1 | 2 | 3 | 4 | 5 |
| 12 | Value own role in preventing patient safety incidents | 1 | 2 | 3 | 4 | 5 |
| 13 | Health care professionals should involve in design, selection, implementation, and evaluation of information technologies to improve patient safety | 1 | 2 | 3 | 4 | 5 |
| 14 | A standardized procedure minimizes risks associated with hand off (e.g., transfer, shifts) within disciplines and across transitions in care | 1 | 2 | 3 | 4 | 5 |

**II. Patient safety skill**

| No. | Survey question | Strongly disagree | Disagree | Neutral | Agree | Strongly agree |
| --- | --- | --- | --- | --- | --- | --- |
| 1 | Report errors using a patient safety reporting system | 1 | 2 | 3 | 4 | 5 |
| 2 | Analyze a case to find the causes of patient safety incidents | 1 | 2 | 3 | 4 | 5 |
| 3 | Support and advise a peer who must decide how to respond to patient safety incidents | 1 | 2 | 3 | 4 | 5 |
| 4 | Disclose an error to a faculty member | 1 | 2 | 3 | 4 | 5 |
| 5 | Communicate observations or concerns related to patient safety incidents with peers or team members | 1 | 2 | 3 | 4 | 5 |
| 6 | Communicate observations or concerns related to patient safety incidents with an affected patient and his or her family | 1 | 2 | 3 | 4 | 5 |
| 7 | Use technology and information management tools (e.g., bar codes, electronic medical record, automatic alerts and alarms, Drug Utilization Review) to support safe processes of care | 1 | 2 | 3 | 4 | 5 |
| 8 | Practice hand hygiene to prevent infection | 1 | 2 | 3 | 4 | 5 |
| 9 | Administer drug to patient according to medication policies for safe care | 1 | 2 | 3 | 4 | 5 |
| 10 | Follow communication practices that minimize risks associated with hand offs between and among providers and across transitions in care | 1 | 2 | 3 | 4 | 5 |
| 11 | Use standard infection control precautions for all patient encounters and other transmission precautions as appropriate | 1 | 2 | 3 | 4 | 5 |
| 12 | Check patient’s identity accurately (e.g., a registration number, birth date, name) | 1 | 2 | 3 | 4 | 5 |

**III. Patient safety knowledge**

| No. | Survey question | Strongly disagree | Disagree | Neutral | Agree | Strongly agree |
| --- | --- | --- | --- | --- | --- | --- |
| 1 | Describe factors that create a culture of safety (e.g., teamwork, leadership, effective communication) | 1 | 2 | 3 | 4 | 5 |
| 2 | Describe role of human factors in assuring safety (e.g., physical, psychological limitations of human, interactions between human and instrument) | 1 | 2 | 3 | 4 | 5 |
| 3 | Describe definition of near misses | 1 | 2 | 3 | 4 | 5 |
| 4 | Describe definition of adverse events | 1 | 2 | 3 | 4 | 5 |
| 5 | Describe definition of sentinel events | 1 | 2 | 3 | 4 | 5 |
| 6 | Describe processes used in analyzing causes of patient safety incidents (e.g., root cause analysis) | 1 | 2 | 3 | 4 | 5 |
| 7 | Describe the impact (benefits and limitations) of technology and information management care (e.g., bar codes, electronic medical record, automatic alerts and alarms, Drug Utilization Review) on patient safety | 1 | 2 | 3 | 4 | 5 |
| 8 | Explain how authority gradients (horizontal, vertical) influence teamwork and patient safety | 1 | 2 | 3 | 4 | 5 |
